# Targeting nonsense-mediated mRNA decay in colorectal cancers with microsatellite instability

**DOI:** 10.1038/s41389-018-0079-x

**Published:** 2018-09-19

**Authors:** A’dem Bokhari, Vincent Jonchere, Anaïs Lagrange, Romane Bertrand, Magali Svrcek, Laetitia Marisa, Olivier Buhard, Malorie Greene, Anastasia Demidova, Jieshuang Jia, Eric Adriaenssens, Thierry Chassat, Denis S. Biard, Jean-François Flejou, Fabrice Lejeune, Alex Duval, Ada Collura

**Affiliations:** 1 Sorbonne Université, UPMC Univ Paris 06, INSERM, UMRS 938, SIRIC CURAMUS, Equipe Instabilité des Microsatellites et Cancer, Equipe Labellisée par la Ligue Contre le Cancer, Centre de Recherche Saint Antoine, Paris, F-75012 France; 2 AP-HP, Hôpital Saint-Antoine, Service d’Anatomie et Cytologie Pathologiques, Paris, France; 3 Programme “Cartes d’Identité des Tumeurs”, Ligue Nationale Contre le Cancer, Paris, France; 4 Univ. Lille, CNRS, Institut Pasteur de Lille, UMR 8161—M3T—Mechanisms of Tumorigenesis and Target Therapies, F-59000 Lille, France; 5 INSERM U908, Cell Plasticity and Cancer, University of Lille, 59655 Villeneuve d’Ascq, France; 6 Pasteur de Lille—PLEHTA (Plateforme d’expérimentation et de Haute Technologie Animale), 59019 Lille, France; 7 Centre d’Etude Atomique, Direction des Sciences du Vivant, Institut des Maladies Emergentes et des Thérapies Innovantes, Service d’Etude des Prions et des Infections Atypiques, Fontenay-aux-Roses, France

## Abstract

Nonsense-mediated mRNA decay (NMD) is responsible for the degradation of mRNAs with a premature termination codon (PTC). The role of this system in cancer is still quite poorly understood. In the present study, we evaluated the functional consequences of NMD activity in a subgroup of colorectal cancers (CRC) characterized by high levels of mRNAs with a PTC due to widespread instability in microsatellite sequences (MSI). In comparison to microsatellite stable (MSS) CRC, MSI CRC expressed increased levels of two critical activators of the NMD system, UPF1/2 and SMG1/6/7. Suppression of NMD activity led to the re-expression of dozens of PTC mRNAs. Amongst these, several encoded mutant proteins with putative deleterious activity against MSI tumorigenesis (e.g., HSP110DE9 chaperone mutant). Inhibition of NMD in vivo using amlexanox reduced MSI tumor growth, but not that of MSS tumors. These results suggest that inhibition of the oncogenic activity of NMD may be an effective strategy for the personalized treatment of MSI CRC.

## Introduction

Microsatellite instability (MSI) colorectal cancers (CRC) are a subset of CRC characterized by defects in the DNA mismatch repair (MMR) system. MMR is a system for recognizing and repairing erroneous insertion, deletion, and misincorporation of bases that can arise during DNA replication and recombination, as well as for repairing some forms of DNA damage^1^. Inactivation of the MMR system is not in itself a direct transforming event, with additional genetic changes being necessary for MMR-deficient cells to become malignant. Mutations in human MMR proteins affect genomic stability, leading to widespread alterations in the length of microsatellite repeat sequences in the genome. The MSI phenotype was first observed in tumors from patients with Lynch syndrome (for review see ref. ^2^), and later in sporadic cases of colon, gastric, and endometrial cancer. The highest incidence of MSI tumors occurs in epithelial cancers, but low frequencies of MSI have also been reported in many other cancer types^3^.

In MMR-deficient tumor cells, DNA polymerase slippage at coding sequences can induce frameshift mutations (1 or 2 bp insertion/deletion) that results in the production of truncated proteins. Over the past 20 years, hundreds of different frameshift-truncating mutations have been reported in genes involved in various biological pathways such as cell cycle regulation (e.g., *TGFBR2, IGFR2, TCF4, AXIN2, PTEN, RIZ*), apoptosis (e.g., *BAX, CASP5, BLC10, APAF1, FAS*), DNA damage repair and DNA integrity checkpoint systems (e.g.*, ATR, DNA-PK, RAD50, MSH3, MSH6, MBD4, MLH3, BLM, CHK1*) amongst others (for review see ref. ^2^). By affecting microsatellites located proximally in the cDNA, most frameshift mutations result in the loss of tumor suppressor-related functions. In some cases, however, frameshift mutations can alter coding repeats that are located down-stream of important functional gene domains, or up-stream of other regulatory sites. In these latter contexts, such mutations are proposed to have a dominant negative effect, as for example with the Axin gene^4^. In others, they are thought to be activating mutations, as proposed for the *TCF-4* gene. Truncation mutations of *TCF-4* in MSI cancers enhance the transactivating properties of this transcription factor by favoring the synthesis of isoforms that have lost their capacity to bind CtBP, a transcriptional repressor of the TCF/LEF family^5^.

Nonsense-mediated decay (NMD) is a ubiquitous surveillance mechanism that recognizes and degrades transcripts containing premature termination codon (PTC)^6^. A crucial aspect of the NMD pathway is the ability to distinguish normal termination codons from PTCs. The discrimination of PTC-containing mRNA in mammalian cells depends on the PTC position in the mRNA. mRNAs with a PTC located in the coding sequence excluding in the last exon, or more than 55 bp up-stream of the last exon–exon junction, are recognized as NMD substrates^7^. In contrast, PTCs located down-stream of the last exon–exon junction are not degraded by the NMD system^8,9^. The central component of the NMD pathway in mammalian cells is the protein UPF1. Activation of NMD leads to the transition of the SURF surveillance complex, consisting of the UPF1, SMG1, eRF1, and eRF3 factors, to the decay-inducing complex (DECID). The latter is formed following interaction of the SURF complex with UPF2, UPF3b, and an exon junction complex (EJC) down-stream of the PTC. Remodeling of NMD complexes occurs following the phosphorylation of UPF1 by the protein kinase SMG1^10^. Phosphorylated UPF1 proteins associate with the endonuclease proteins SMG5, SMG6, and SMG7^11^, and with general mRNA degradation factors such as POP2, leading to mRNA degradation^12^.

Approximately 30% of genetic disorders are due to non-sense or frameshift mutations that lead to PTC mRNAs encoding mutant proteins with no residual activity^13,14^. However, there are also examples where NMD degrades mutant proteins that have some residual activity. In such cases, NMD could have beneficial or detrimental effects depending on the mutation and on the disease^15,16^. In cancer, NMD can enhance the defects caused by frameshift mutations associated with partial loss of function in tumor suppressor genes^15,17,18^. Because of the high number of PTC-mRNAs generated by MSI in MMR-deficient neoplasms, NMD can halt the expression of many endogenous mutants, some of which may have a role in cancer development^19–21^. In MSI CRC, we and others have already highlighted the relevance of NMD in MSI-driven mutant mRNAs and in particular the important role of UPF1 in this process^20^. Despite its potential clinical relevance, the role of NMD in MSI tumorigenesis has not been investigated, possibly due to the lack of effective inhibitors of this system. In the present study, we used recently developed NMD inhibitors to target this system in MSI and MSS models of CRC. Our results highlight the important oncogenic impact of NMD on MSI-driven tumorigenesis and suggest that NMD inhibition may be a novel and effective strategy for the personalized treatment of MSI CRC.

## Results

### NMD factors are overexpressed in MMR-deficient primary colon tumors and NMD activity causes the decay of hundreds of MSI-driven PTC mRNAs

**Fig. 1:**
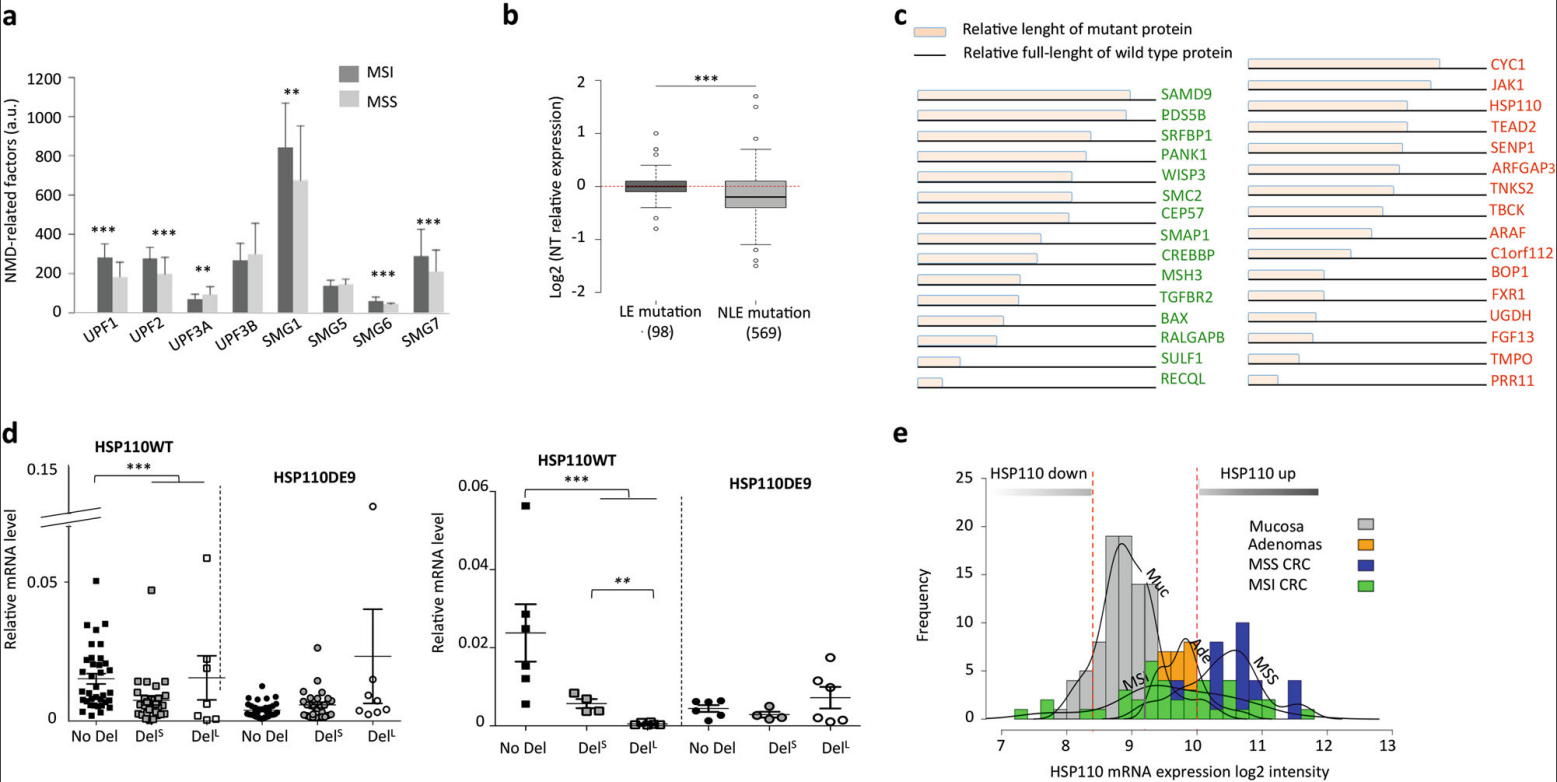
The overexpression of NMD factor in MSI primary CRC. **a** Microarray analysis of NMD-related factors in MSI/MSS tumor tissues ([MSS primary CRC], *n* = 48; [MSI primary CRC], *n* = 40). **b** Microarray analysis associated with Exome sequencing data of 30 MSI tumors to compare the expression of genes with mutation in microsatellite located in the last exon (LE; *n* = 98) or before the last exon (NLE; *n* = 569). **c** Schematic structure of mutant proteins (15 mutated tumor suppressor genes in green or 16 mutated oncogenes in red) with NLE mutations and significant down-regulation in MSI tumors. **d** Left panel: relative expression levels of HSP110wt and HSP110DE9 mRNAs by quantitative RT-PCR in MSS and MSI CRC primary tumors. No Del (wild type status; MSS CRCs) *n* = 36; DelS (small deletion: ≤4 pb; MSI CRCs), *n* = 28; DelL (large deletion: >4 pb; MSI CRCs), *n* = 7. Right panel: relative mRNA expression levels of HSP110wt and HSP110DE9 determined by quantitative RT-PCR in MSS and MSI CRC cell lines. No T17 deletion [No Del], *n* = 6 CRC cell lines (ISI, SW1116, V9P, ALA, FET, SW480); small T17 Deletion [DelS], *n* = 4 CRC cell lines (HCT8, HCT116, TC71, LIM1215); large T17 Deletion [DelL], *n* = 6 CRC cell lines (TC7, Lovo, KM12, LS411, Ls174T, Co115). Data are means ± SEM. **e** Quantification of all HSP110 mRNAs. Densities and bar plots of HSP110 log2 intensities in normal colonic mucosa (Muc), adenomas (Ade), MSS tumors (MSS), and MSI tumors (MSI). All Data are means ± SEM. Unpaired *t*-test was performed to determine significance. **p < 0.01, ***p < 0.001, ****p < 0.0001

Expression levels for NMD-related factors (*UPF1*, *UPF2*, *UPF3A*, *UPF3B*, *SMG1*, *SMG2*, *SMG6*, and *SMG7*) were calculated from microarray-derived datasets of primary tumor samples (*n* = 40 MSI, *n* = 48 MSS) and matched normal colon samples (*n* = 95). The expression level of *UPF1* and of other NMD-related factors was higher in primary MSI CRC vs MSS CRC (Fig. 1a). Consistent with its negative effect on NMD activity^22^, *UPF3A* expression was significantly down-regulated in MSI vs MSS primary tumors.

Exome sequencing of 30 MSI CRC from this cohort allowed us to identify MSI-driven somatic events within coding repeats (unpublished data). Significant down-regulation of mutant mRNA was observed for genes with mutations in coding repeats that were located before the final exon (near last exon, NLE; *n* = 569). This was not observed for mutant transcripts from genes harboring mutations contained within the final exon (last exon, LE; *n* = 98) (Fig. 1b, ***p-value = 1E−06), indicating decay of these mRNA mutants in MSI tumor cells.

### Down-regulation of HSP110DE9 and other mutant mRNAs that encode cancer-related proteins with residual activities in MSI CRC

From the 569 genes with NLE mutations, further studies were carried out on the 71 mutated MSI target mRNAs that displayed significant down-regulation (fold-change < −0.5) in MSI CRC (Supplementary Figure S1). These 71 genes included target genes for MSI with cancer-related functions (15 tumor suppressor-like genes and 16 oncogenes) and in which the frameshift mutated proteins may have retained complete or partial biological activity. Examples include the tumor suppressor genes *WISP3*^23^, *PANK1*^24^ and *SRFBP1*^25^, and the oncogene *JAK1*^26,27^ (Fig. 1c).

Of note, the HSP110 chaperone protein in MSI tumors is frequently altered by frameshift mutations. In these tumors, HSP110DE9 mRNA mutant is produced following bi-allelic deletions in the T17 noncoding DNA repeat of *HSP110* intron 8^28,29^. Small deletions (≤4 bp) of T17 do not induce aberrant splicing of exon 9, whereas large deletions (>4 bp) induce aberrant exon 9 skipping of the HSP110 gene. This leads to an HSP110DE9 mRNA containing a PTC before the last exon, which is therefore sensitive to mRNA decay. *HSP110* was found in the list of MSI target genes with significant mRNA downregulation in MSI CRC (Fig. 1c). Using quantitative RT-PCR, the expression of *HSP110* wild type mRNA was found to be significantly lower in MSI CRC cell lines and primary tumors displaying large T17 deletions (Fig. 1d). In contrast, the expression of *HSP110DE9* mutant transcript was not increased in these samples. Micro-array analyses performed on a larger series of CRC confirmed the lower *HSP110* wild type mRNA level in MSI CRC compared to MSS CRC (Fig. 1e). Similar to other frameshift mutant mRNAs encoding aberrant oncoproteins with residual biological activity (Fig. 1c), we postulate that *HSP110DE9* produces a dominant negative isoform of HSP110 that is strongly degraded by NMD in MSI CRC cells (Fig. 1c–e).

### Inhibition of NMD in colorectal cancer cells and in mouse models can be achieved successfully using commercial NMD inhibitors

**Fig. 2:**
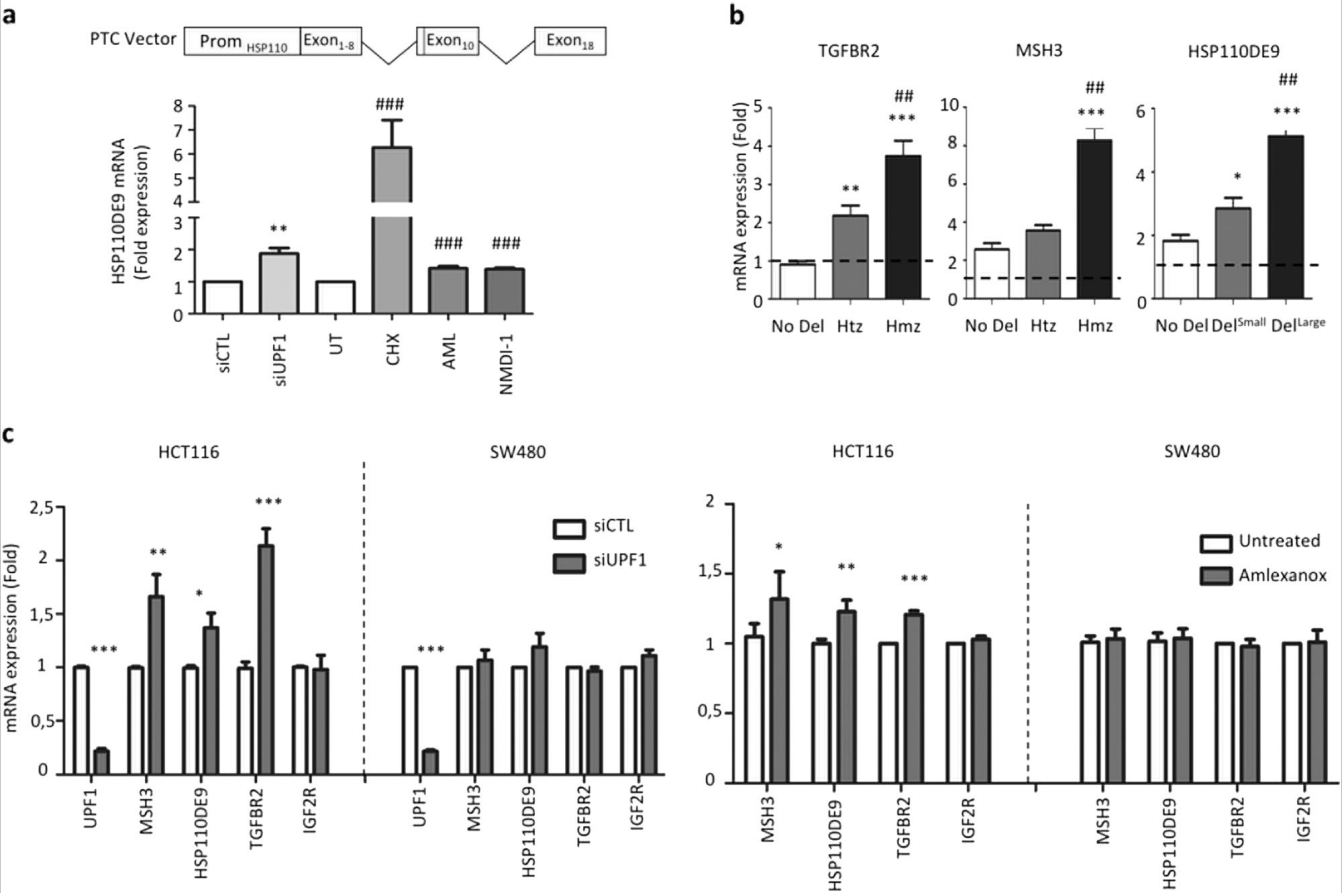
Inhibition of NMD system and differential RNA decay. **a** Upper panel: schematic representation of the HSP110DE9-specific NMD reporter system used in this work. The NMD.reporter gene consisted of an in-frame HSP110 construction and contains the cDNA sequence from exon 1 to exon 8, intron 9, exon 10, intron 16, and exon 18. As in the case of the T_17_ mutation of HSP110 located near the splice acceptor site of intron 8, a nonsense mutation appears in exon 10 due to the frameshift mutation caused by skipping of exon 9, making the exogenous mRNA a target of NMD. This construction is placed in an EBV stable vector. Lower panel: relative expression of HSP110DE9-PTC mRNA from NMD reporter stably transfected in the SW480 CRC cell line treated with several NMD inhibitors (siUPF1, cycloheximide (CHX), amlexanox, and NMDI-1). **b** Relative expression levels of *TGFBR2*, *MSH3*, or *HSP110DE9* mRNAs determined by quantitative RT-PCR in CRC cell lines. After cycloheximide [CHX] treatment (4 h, 400 μg/ml). RNA expression levels compared to untreated cells [UT] in cell lines were analyzed with the *TGFBR2* probe, [No Del] (SW480 and FET), Heterozygote [Htz] (HCT8 and RKO), Homozygote [Hmz] (HCT116 and LS174T); with the *MSH3* probe, [No Del] (SW480 and HCT8), Heterozygote [Htz] = (LS174T), Homozygote [Hmz] (HCT116); with *HSP110DE9* probes, [No Del] (SW480 and FET); [Del^S^] (HCT116 and HCT8); [Del^L^] (RKO and LS174T). Dashed line refers to the ratio calculated between treated and untreated cells with CHX. **c** Relative mRNA expression levels of MSI target genes (containing coding DNA repeats) as determined by quantitative RT-PCR in HCT116 (MSI) and SW480 (MSS) CRC cell lines transfected with siUPF1 (24 h post-transfection; top panel) or treated for 24 h with 5 µM amlexanox (lower panel). *IGF2R* is used as an internal control (not mutated in HCT116 and SW480 CRC cell line) for experimental conditions. All data are means ± SEM. Unpaired *t*-test was performed to determine significance. *p < 0.05, **p < 0.01, ***p < 0.001, ****p < 0.0001

CRC cell lines were treated with two commercially available NMD inhibitors: (i) amlexanox, for which the exact mechanism of action is still unknown^30^, and (ii) NMDI-1, which inhibits the dephosphorylation of UPF1 and thereby keeps it in a non-active state^31^. Two controls were used: an siRNA against the key NMD activator UPF1, and the protein synthesis inhibitor cycloheximide (CHX). As shown in Fig. 2a, treatment with these four agents all resulted in significant re-expression of the exogenous *HSP110DE9* transcript following transfection of MSS SW480 CRC cell lines with a plasmid specially designed for this purpose.

A panel of CRC cell lines was also used to study the differential decay of PTC-mRNAs resulting from MSI-driven mutations. Treatment with CHX increased the expression of mutant PTC-mRNAs derived from MSI target genes (e.g., *TGFBR2*, *MSH3*, or *HSP110*) in MSI vs MSS CRC cell lines (Fig. 2b). Re-expression of *HSP110DE9* mRNA was demonstrated using siRNA targeted to the major NMD factor UPF1, and following amlexanox treatment of the MSI CRC cell line HCT116. UPF1 depletion and amlexanox treatment also led to significant increases in the expression of other mutated PTC-containing transcripts (*TGFBR2, MSH3*), but not of a wild type mRNA control (*IGFR2*). As expected, the levels of endogenous mutant mRNA in the MSS CRC cell line SW480 were not altered following inhibition of NMD (Fig. 2c).

### Inhibition of NMD leads to decreased MSI CRC cell proliferation and has an antitumor effect on MSI tumor xenografts

**Fig. 3:**
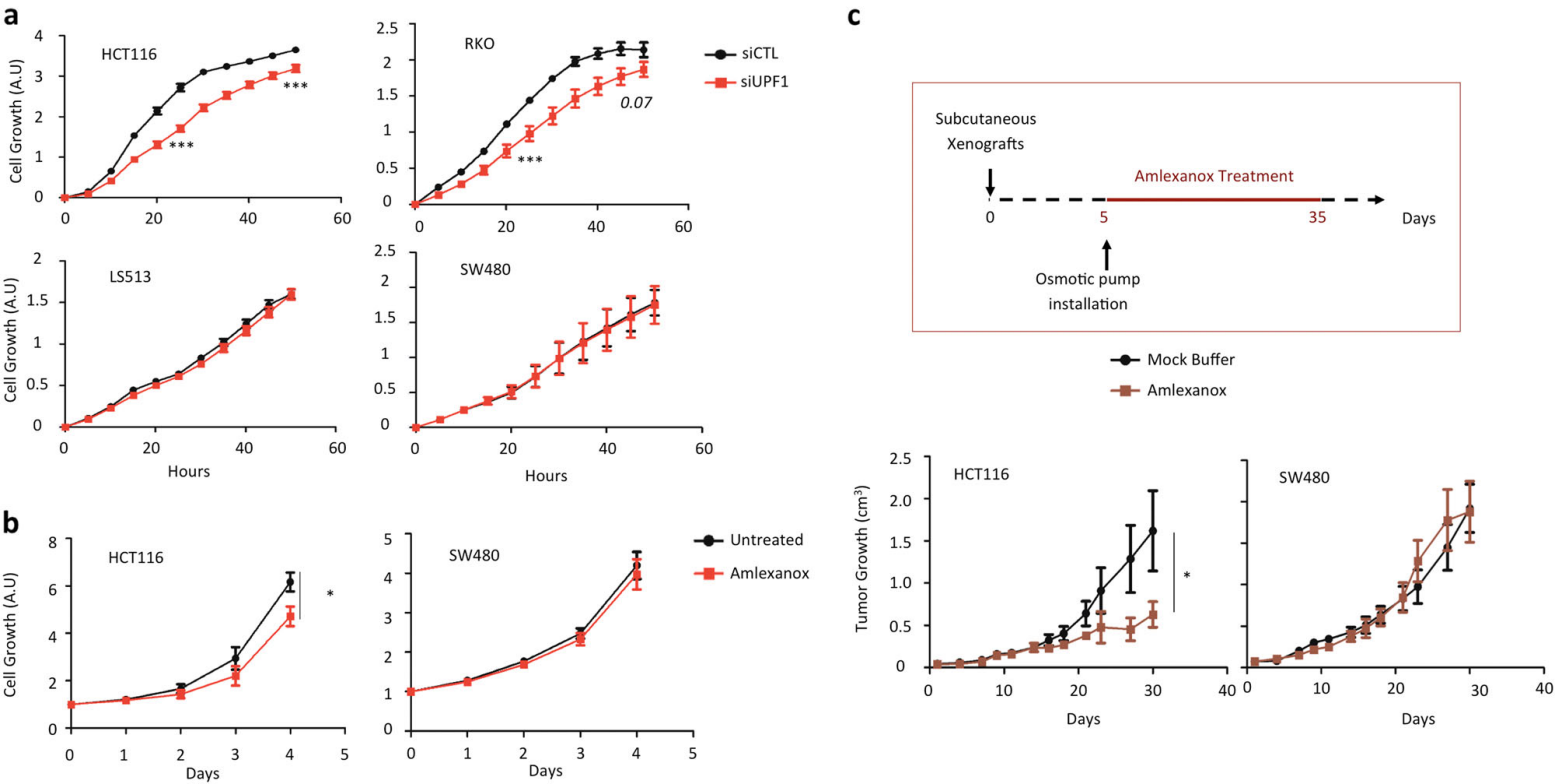
Impact of NMD inhibition in cell proliferation and tumor growth. **a** Cell proliferation of HCT116 (MSI), RKO (MSI), SW480 (MSS), and LS513 (MSS) colon cancer cells were analyzed with Xcelligence technology (see Materials and methods). Twenty-four hours post-transfection with a control (siCTL) or siRNA against UPF1 (siUPF1), cells were seeded in E-plate 96 to allow measurement of proliferation rates during 48 h. **b** HCT116 (MSI) and SW480 (MSS) CRC cancer cells were treated once a day during 4 days with or without 5 μM amlexanox. OD was measured every day after drug treatment using the WST-1 assay. **c** Upper panel: schematic representation of the protocol for treating mice with the NMD inhibitor amlexanox. The osmotic pump contained either a mock buffer made with 50% DMSO and 50% PEG400, or amlexanox diluted in the mock buffer in order to deliver 0.15 mg of amlexanox per day to each mouse during 28 days. Lower panel: comparative analysis of tumor growth (mean tumor volumes) in mice treated with or without amlexanox. Eight mice per group. Experiments were performed with MSI (HCT116) or MSS (SW480) CRC cells (left and right panels, respectively). All data are means ± SEM. Unpaired *t*-test was performed to determine significance. *p < 0.05, ***p < 0.001

The above results suggest that NMD is responsible for the decay of hundreds of PTC-mRNAs in MSI CRC cells, including several that encode cancer-related mutant proteins with residual activity such as *HSP110DE9*. We next compared the functional consequences of NMD inhibition between MSI and MSS cell lines. These are characterized by relatively high and low numbers of PTC-mRNAs, respectively. Inhibition of UPF1 caused decreased cell proliferation in the MSI CRC cell lines (HCT116 and RKO), whereas no effect was seen in the MSS cell lines (LS513 and SW480) (Fig. 3a and Fig. S2). Similarly, amlexanox treatment decreased the cell proliferation rate in HCT116 cells (MSI) but not in SW480 cells (MSS, Fig. 3b).

NMD inhibition using amlexanox led to a significant antitumor effect in xenografts of MSI CRC cells (HCT116) grown in nude mice when compared to treatment with control buffer only (Fig. 3c). However, this effect was not observed in nude mice xenografted with MSS CRC cells (SW480).

## Discussion

In this study, we report the specific overexpression of essential NMD factors such as UPF1/2 and SMG1/6/7 in MSI primary CRC compared to MSS primary CRC. Furthermore, NMD inhibition was shown to adversely affect MSI cell proliferation but not MSS cell proliferation in vitro, and to inhibit MSI tumor growth but not MSS tumor growth in a xenograft model. From a mechanistic standpoint, these results suggest that NMD activity has an oncogenic impact on tumorigenesis in MSI CRC. We have identified a role for NMD in the down-regulation of confirmed or putative toxic mutant proteins that derive from PTC-mRNAs, such as HSP110DE9^28,29^ and others. Our data also show that NMD inhibition using amlexanox, a known inhibitor of NMD^30^, was also efficient in a mouse xenograft model. These in vivo results suggest that NMD could be an interesting target for inhibition in the treatment of patients with MSI colon cancer or other MMR-deficient primary tumors.

**Fig. 4:**
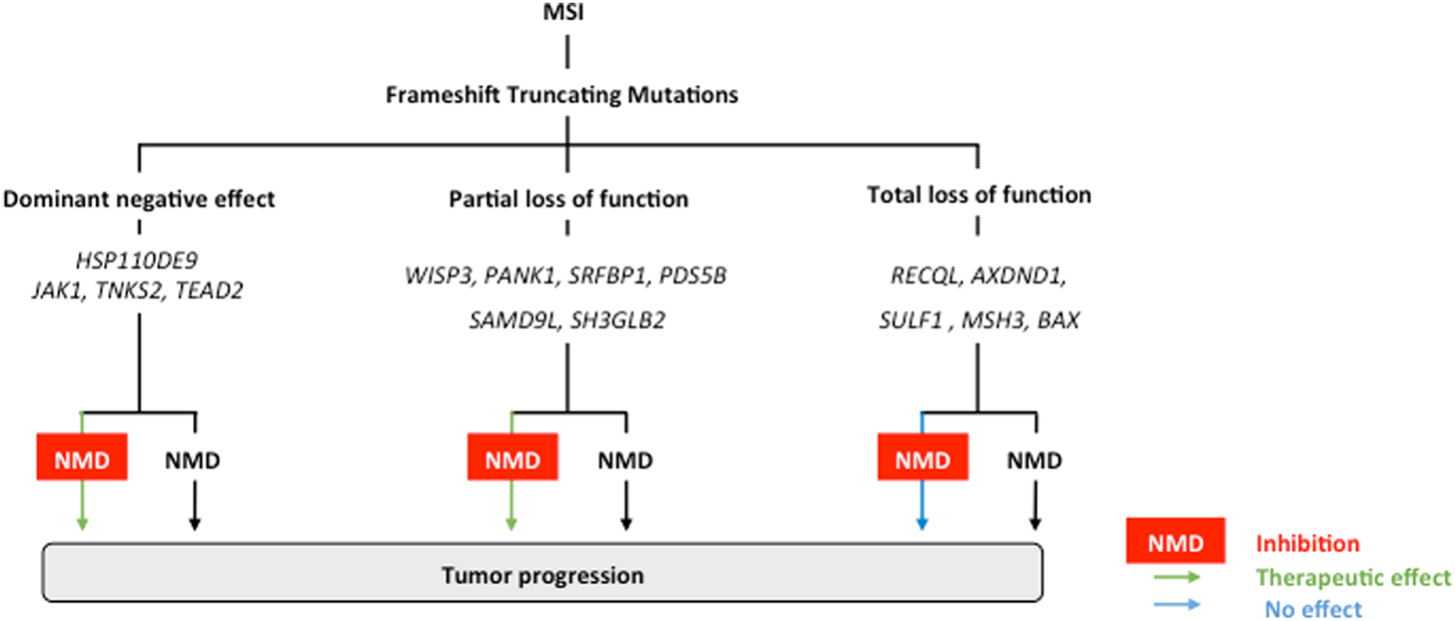
Schematic representation of the role of NMD in MSI tumors and the expected therapeutic benefit of NMD blockade in this tumor model. Numerous MSI-driven mutant PTC-mRNAs processed by NMD are generated due to frameshift-truncating mutations in coding DNA microsatellites in MMR-deficient colon tumors. These mutations usually result in loss of function effects and they inactivate tumor suppressor genes (e.g., *BAX*, *MSH3*…). Although the corresponding mutant PTC-containing mRNAs are degraded by NMD or not it is of poor functional significance when the mutant proteins have totally lost their function and therefore do not have residual biological activity. In contrast, it is expected that inhibiting NMD could be deleterious for MSI cancer cells when mutant mRNAs encode for (i) tumor-suppressor mutant proteins that have retained some residual activity (notably, partial loss of function is expected when the truncation occurs down-stream of important functional domains in the protein), or (ii) dominant negative mutant proteins like HSP110DE9

In previous publications, we and others have proposed that NMD may play a role during MSI tumorigenesis^20,32^. Nevertheless, this putative role was poorly understood and thought to be quite complex depending on the gene function for the mutation targeted by NMD factors. Previous exome sequencing analysis of 30 MSI primary cancers performed by our group allowed us to comprehensively identify all indel-mutations that lead to PTC formation and are targeted by NMD. Amongst the 71 mutated MSI target genes in which the PTC-mRNAs were expected to be down-regulated by NMD, we identified 15 aberrant transcripts encoding mutant proteins with putative retained tumor suppressor functions, including *PDS5B, WISP3*, and *PANK1*. The PDS5B mutant protein, for example, retains all the regions and residues involved in the recruitment and release of cohesin and other partners during mitosis, suggesting it has conserved tumor suppressor activity in MSI cancer cells^33^. Moreover, we identified 16 aberrant transcripts that encoded oncoproteins, including HSP110, JAK1, ARAF, and TNKS2. As previously reported, HSP110DE9 protein has dominant negative activity and its aberrant mRNA is targeted by NMD. HSP110DE9 interacts with wild type HSP110, thereby strongly inhibiting its oncogenic function^28,29,34^. Another putative dominant negative mutation affects the *ARAF* proto-oncogene in MSI CRC cells. The MSI-driven frameshift mutation of *ARAF* results in a truncated protein due to deletion of 293 aa. This mutant is closely related to the DA-Raf1 dominant-negative mutant, which lacks the kinase domain and is reportedly an antagonist of the Ras-Erk pathway^35^. Therefore, several mechanisms are likely to underpin the oncogenic effects of NMD in MSI CRC and these will require further investigation in future studies. Figure 4 shows the putative role of NMD in MSI tumors and how a therapeutic benefit from NMD inhibition could be expected in this model.

A major finding of this work was the demonstration of efficient inhibition of NMD activity using Amlexanox. Other pharmacological agents have previously been used to inhibit NMD, including cycloheximide^36^, emetine^37^, puromycin^38^, pateamine A^39^, caffeine, wortmannin, and NMDI-1^15,40,31^. However, these are not suitable for clinical use because of their toxicity or weak activity. In contrast, the NMD inhibitor amlexanox is already being used to treat patients with recurrent aphthous stomatitis^41^. We demonstrated here that treatment with amlexanox resulted in significant re-expression of several PTC mRNAs encoding HSP110DE9 and also other toxic mutant proteins. Probably as a consequence of this, MSI cancer cells are particularly sensitive to NMD inhibition. Amlexanox treatment led to significant and specific inhibition of the growth of MSI tumor xenografts in vivo, while showing no apparent toxicity in mice or in humans treated for aphthous ulcers^42^. However, it should be noted that amlexanox has been reported to inhibit TBK1 and IKK-ε protein kinases in mice^43^ and could therefore have other effects unrelated to NMD inhibition, as found here. Moreover, although amlexanox is already in clinical use, it is clear that it was not a strong inhibitor of NMD in our hands. In future studies, we plan to use other agents that could be more powerful inhibitors of NMD in tumor cells. Interestingly, such agents could also be of therapeutic interest since they are expected to enhance T cell antitumor immunity in MSI CRC by favoring the presentation of numerous MSI-driven aberrant neoantigens at the surface of tumor cells. Whether NMD inhibition could be used synergistically with immune checkpoint inhibitors that are currently being trialed in patients with MSI cancer remains to be determined.

## Materials and methods

### CRC cell lines and clinical tumor samples

CRC cell lines were obtained from the American Type Culture Collection and were grown in DMEM containing 10% fetal bovine serum and antibiotics. They were free of mycoplasma. Tissue samples of primary tumor and normal colon were obtained from patients undergoing surgery for CRC (Hôpital Saint-Antoine, Paris, France). The MSI status of tumors was assessed as reported previously^44^.

### Analysis of *HSP110* T17 deletion and real-time quantitative RT-PCR

The QIAmp DNA Mini Kit (Qiagen) was used to extract DNA. The deletion status of the T17 repeat in *HSP110* was determined as previously described^45^. Total RNA was obtained using the RNeasy Mini kit (Qiagen). The integrity of RNA (RIN) from all primary tumor samples was assessed on a 2100 Bioanalyzer using the RNA 6000 Nano LabChip kit (Agilent). Only those with RIN of >5 were evaluated further. Complementary DNA was made using a High Capacity cDNA reverse transcription kit (Applied Biosystems) and the Applied SDS Biosystems analysis software used for quantitative RT-PCR. *HSP110wt* and *HSP110DE9* transcript levels were calculated relative to RPLP0 ubiquitous RNA, while *TGFBR2, MSH3, BAX, IGF2R*, and *GAPDH* transcript levels were calculated relative to 18S ubiquitous RNA. The primers and probes for *HSP110wt* and *HSP110DE9* were reported previously^45^. Primers and internal probes for *TGFBR2, MSH3, BAX, IGF2R, GAPDH, 18S*, and *RPLP0* were those recommended by Applied Biosystems (TaqMan gene expression assays). The cycling conditions for PCR consisted of an initial denaturation step at 95 °C (10 min duration) followed by 40 cycles of 95 °C (15 s) and 60 °C (1 min).

### In vitro treatments with cycloheximide, NMDI-1 and amlexanox

Cells were grown in 6-well plates (2 × 10^5^ cells seeded per well) in DMEM media. Cycloheximide (Sigma-Aldrich) was added at 400 μg.ml^−1^ for 4 h, amlexanox (Sequoia Research Products) at 5 μM for 24 h, or NMDI-1 (S.E. Velu Laboratory) at 2.5 μM. Cells were then harvested and the RNA extracted using RNeasy Mini Kit (Qiagen®).

### Transfection with siRNA

HCT116 CRC cells were grown in 6-well plates (1 × 10^5^ cells seeded per well) and then transfected with siRNA (50 nM) targeted against UPF1 or else with non-specific siRNA (DharmaFECT 1, Thermo Fisher). siRNA targeted against GAPDH was used as the control. Cells were harvested for total RNA extraction at 48 h after transfection, with all experiments done in triplicate.

### Cell proliferation

The reagent WST-1 (SIGMA®) was used to quantify cell proliferation. Cells (2 × 10^4^ per well) were seeded into 24-well plates and grown in 2 mL of supplemented DMEM medium. They were treated once each day with amlexanox for 4 days at concentrations of 5 or 25 µM. For the cell proliferation assay, WST-1 reagent was added at each time point and incubation was for 4 h at 37 °C. Absorbance measurements were made at 450 nm and the reference wavelength used was 750 nm. Rates of cell proliferation were evaluated using XCelligence (OZYME® and ACEA Biosciences®). Following transfection with siUPF1, cells were plated in E-plate 96 containing gold sensor arrays (electrodes). Measurement of impedance by the sensor electrodes allows any physiological changes to cells to be monitored.

### Whole-exome sequencing

A series of 30 MSI CRC and paired adjacent normal mucosa were previously sequenced for mutations in microsatellite sequences (https://www.ebi.ac.uk/ega/home; Accession No. EGAS00001002477). The position of mutations in the transcripts was based on RefSeq hg19 annotation. mRNA expression in these tumors was also assessed using microarrays as previously described.

### Measurement of HSP110 and NMD factor mRNA expression

A series of MSI (*n* = 40) and MSS (*n* = 48) CRC, as well as normal colonic mucosa (*n* = 42) were evaluated for mRNA expression using Affymetrix U133Plus chips^46^ (data partially contained within the GSE33582 data set). Additional adenoma and normal mucosa samples were included from the GSE8671 and GSE4183 data sets. Data was normalized (Robust Multi-array Average normalization, R package affy) and associations with variables were estimated using ANOVA or *t*-test (R package stats). Comparison of NMD factor expression between MSI and MSS tumors was made using *t*-test and corrected for multiple testing (R package limma).

### In vivo effect of amlexanox

In the xenograft experiments, approximately 10^7^ cells (SW480 or HCT116 CRC cell lines) were injected subcutaneously into the right flank of 5-week old female nude mice (Charles River Laboratories). After the tumor had grown to about 4 mm, an osmotic pump was placed subcutaneously on the left flank of each animal. These contained either a 50% DMSO/50% PEG400 buffer, or the same buffer containing amlexanox. Tumor growth was determined by caliper measurement 3 times per week for 30 days and blinded to the treatment status. Mice were treated in accordance with the ethical regulations of the French Ministry of Research and Technology.

### HSP110DE9-NMD assays

The HSP110DE9-specific NMD-target vector was made by cloning into an EVB plasmid an insert containing the HSP110 cDNA sequence: exons 1–8, intron 8, exon 10, intron 16, and exon 18. This was under the control of the HSP110 promoter. Both HCT116 and SW480 cells were transfected with this HSP110DE9-specific NMD-target vector using Lipofectamine (Invitrogen). Cells were treated with amlexanox (5 or 25 μM) at 24 h after the start of transfection and 24 h later the cells were harvested for analysis of RNA expression.

## Electronic supplementary material

Figure S1

Figure S2

Supplementary legends
