# Supplementary figures and images for: Targeting nonsense-mediated mRNA decay in colorectal cancers with microsatellite instability
Source: Oncogenesis. 2018 Sep 19;7(9):70. doi: 10.1038/s41389-018-0079-x (PMC6143633; doi:10.1038/s41389-018-0079-x)

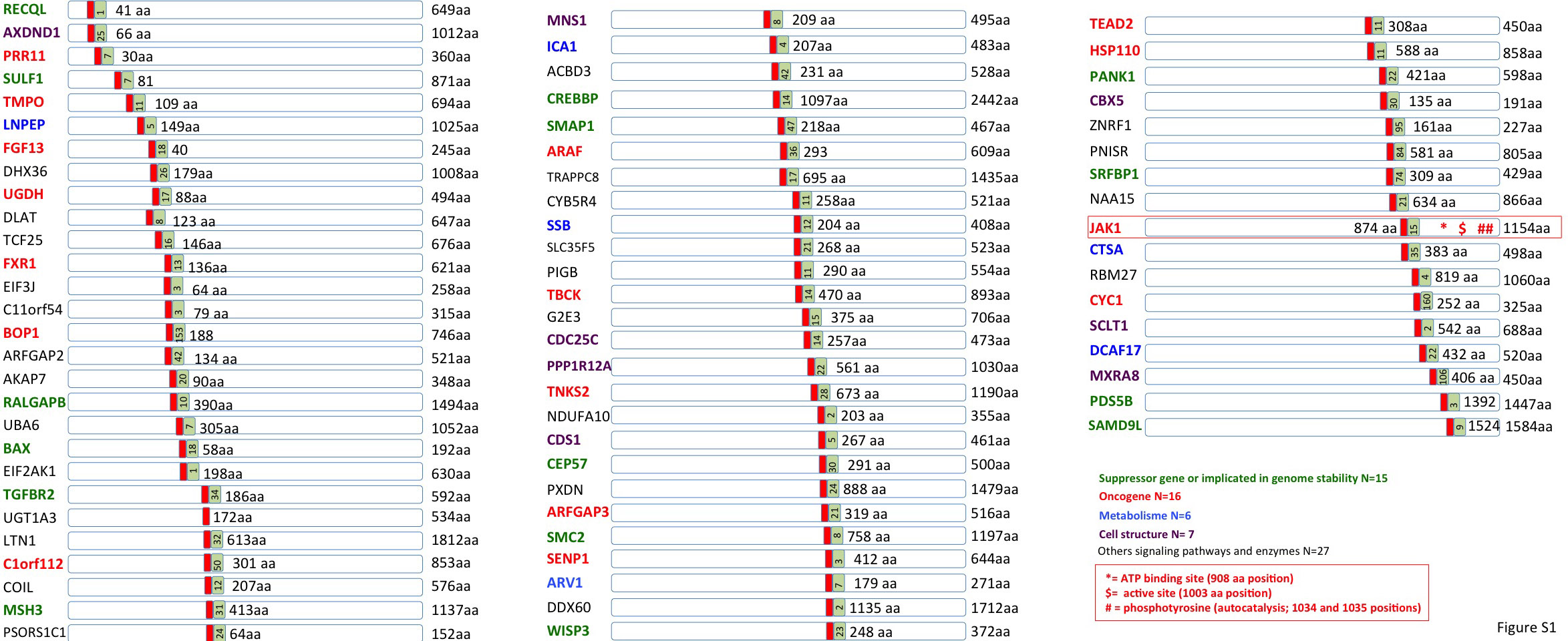

Supplement: Supplementary file 1 — Figure S1 [file 41389_2018_79_MOESM1_ESM.jpg]

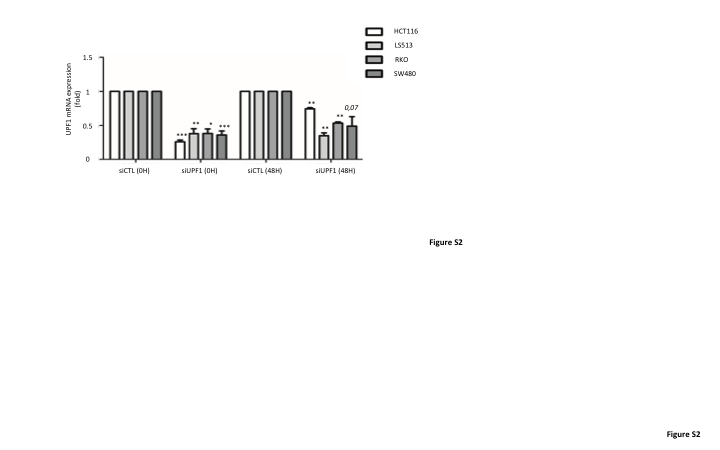

Supplement: Supplementary file 2 — Figure S2 [file 41389_2018_79_MOESM2_ESM.tif]
