# Supplementary material for: Targeting nonsense-mediated mRNA decay in colorectal cancers with microsatellite instability
Source: Oncogenesis. 2018 Sep 19;7(9):70. doi: 10.1038/s41389-018-0079-x (PMC6143633; doi:10.1038/s41389-018-0079-x)
Supplement: Supplementary file 3 — Supplementary legends [file 41389_2018_79_MOESM3_ESM.docx]

**SUPPLEMENTARY LEGENDS**

**S1**: Schematic representation of mutant proteins from the 71 mutated genes with NLE (not last exon) mutations and significantly down-regulated (fold-change < -0.5) in MSI tumors. Position of microsatellite (red square); the size of the neoantigenic sequence is indicated in number of amino acids (green square).

**S2**: Inhibition of UPF1 in MSI (HCT116, RKO) and MSS (SW480, LS513) colon cancer cell lines by RNA interference. Suppression of UPF1 mRNA is measured by quantitative RT–PCR 24h (0H) and 72H (48H) post-transfection compared to non-silencing scrambled control siRNA-transfected cells (siCTL). 18S is used as the reference gene. Data are means ± SEM. Unpaired *t-test* was performed to determine significance. **** indicate: * p <0.05 ** p < 0.01; ***p < 0.001, **** p < 0.0001.
